# Supplementary material for: GR13-type plasmids in Acinetobacter potentiate the accumulation and horizontal transfer of diverse accessory genes
Source: Microb Genom. 2022 Jun 22;8(6):mgen000840. doi: 10.1099/mgen.0.000840 (PMC9455709; doi:10.1099/mgen.0.000840)
Supplement: Supplementary material 1 [file mgen-8-840-s001.pdf]

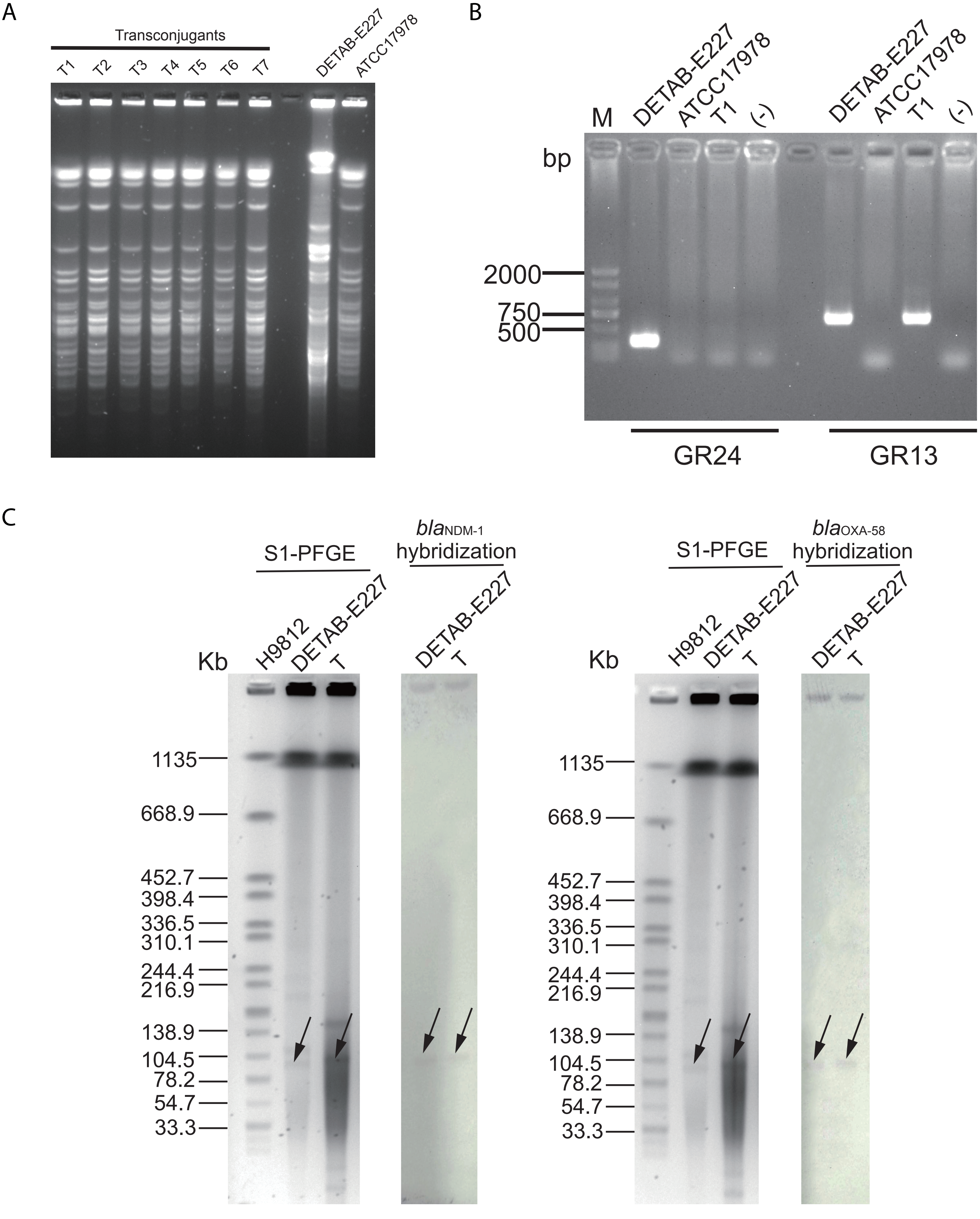

Figure S1: Transfer of pDETAB5. A) ApaI-treated genomic DNA from DETAB-E227, ATCC 17978 and putative ATCC 17978 transconjugants after pulsed-field gel electrophoresis. (PFGE) B) Agarose gel showing the products of PCRs targeting the rep genes of pDETAB4 (GR24) and pDETAB5 (GR13). The source of template DNA for each reaction is labelled above, with (-) indicative of a no-DNA control. The sizes in base pairs of labelled DNA size marker bands are indicated to the left. C) S1-treated DNA after PFGE and hybridisation with bla_{NDM-1} and bla_{OXA-58}-specific probes. The sizes of bands in the DNA size marker (in kilobase pairs) are indicated to the left.

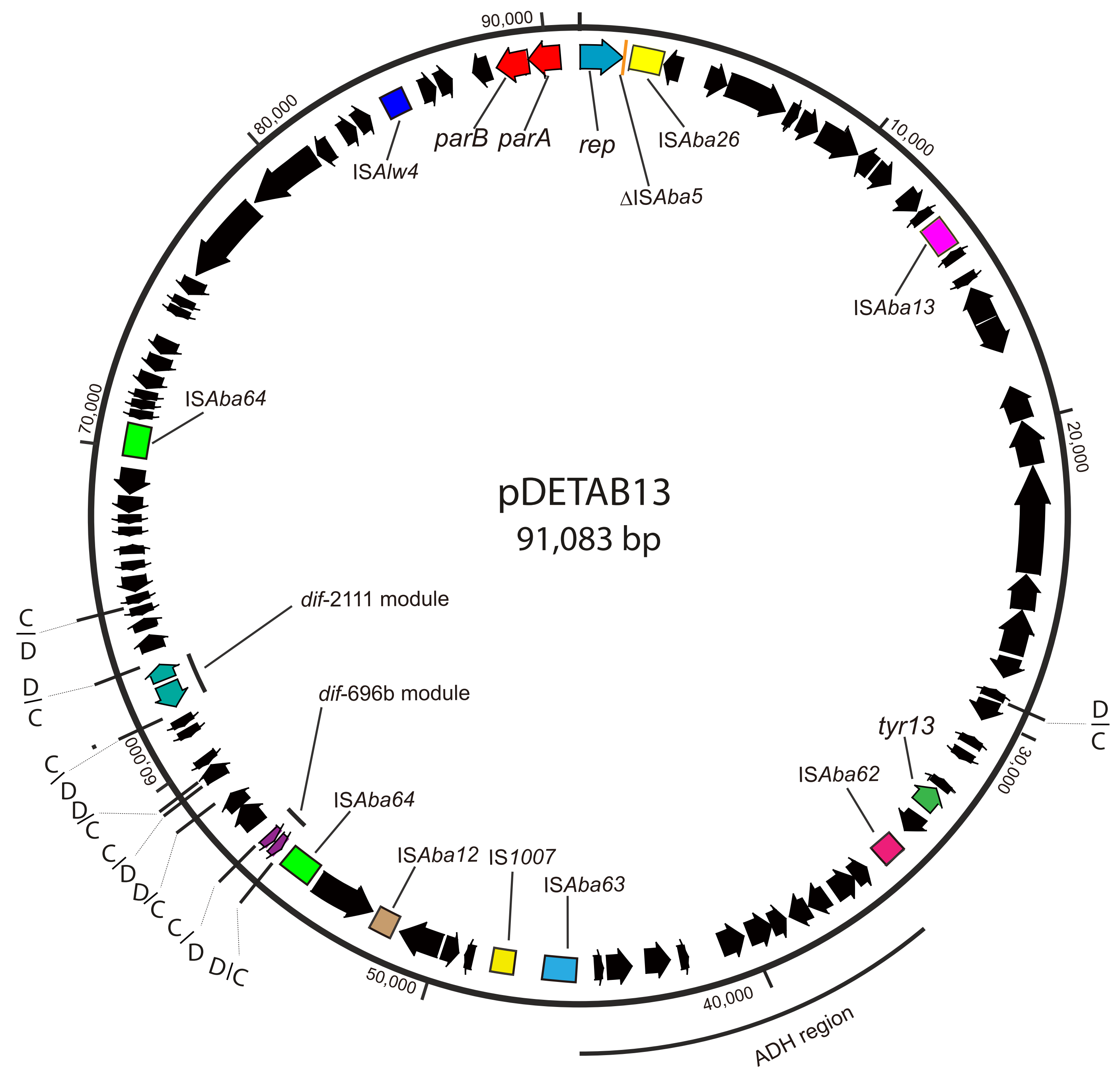

Figure S2: Circular map of pDETAB13. Plasmid sequence is shown as a black line, with arrows inside representing ORFs. Coloured boxes represent IS. Black lines marked "C/D" or "D/C" represent pdif sites and indicate their orientations. Drawn to scale from GenBank accession CP073061.

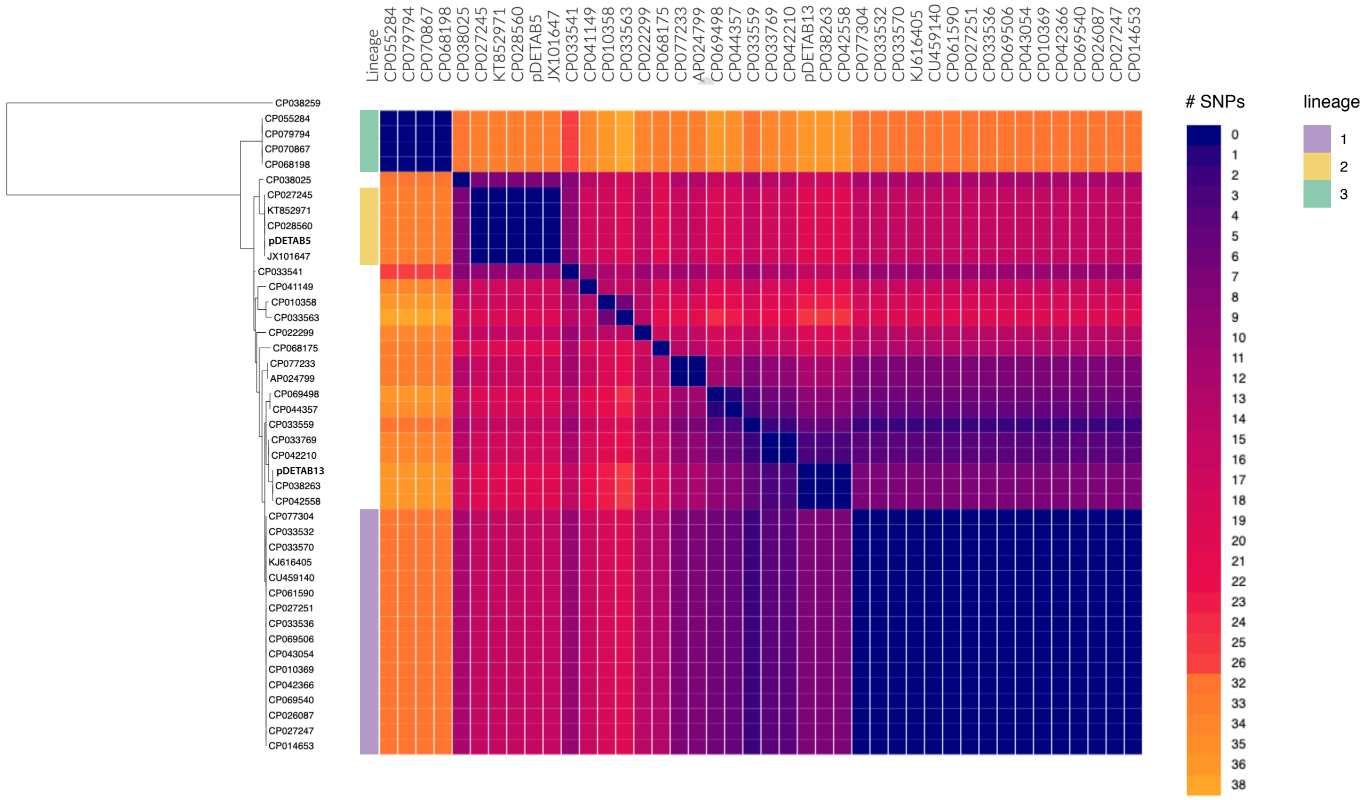

Figure S3: GR13 *rep* gene SNP matrix. The numbers of SNPs between *rep* genes and plasmid lineage memberships are indicated by shading as outlined in the legends to the right of the grid.

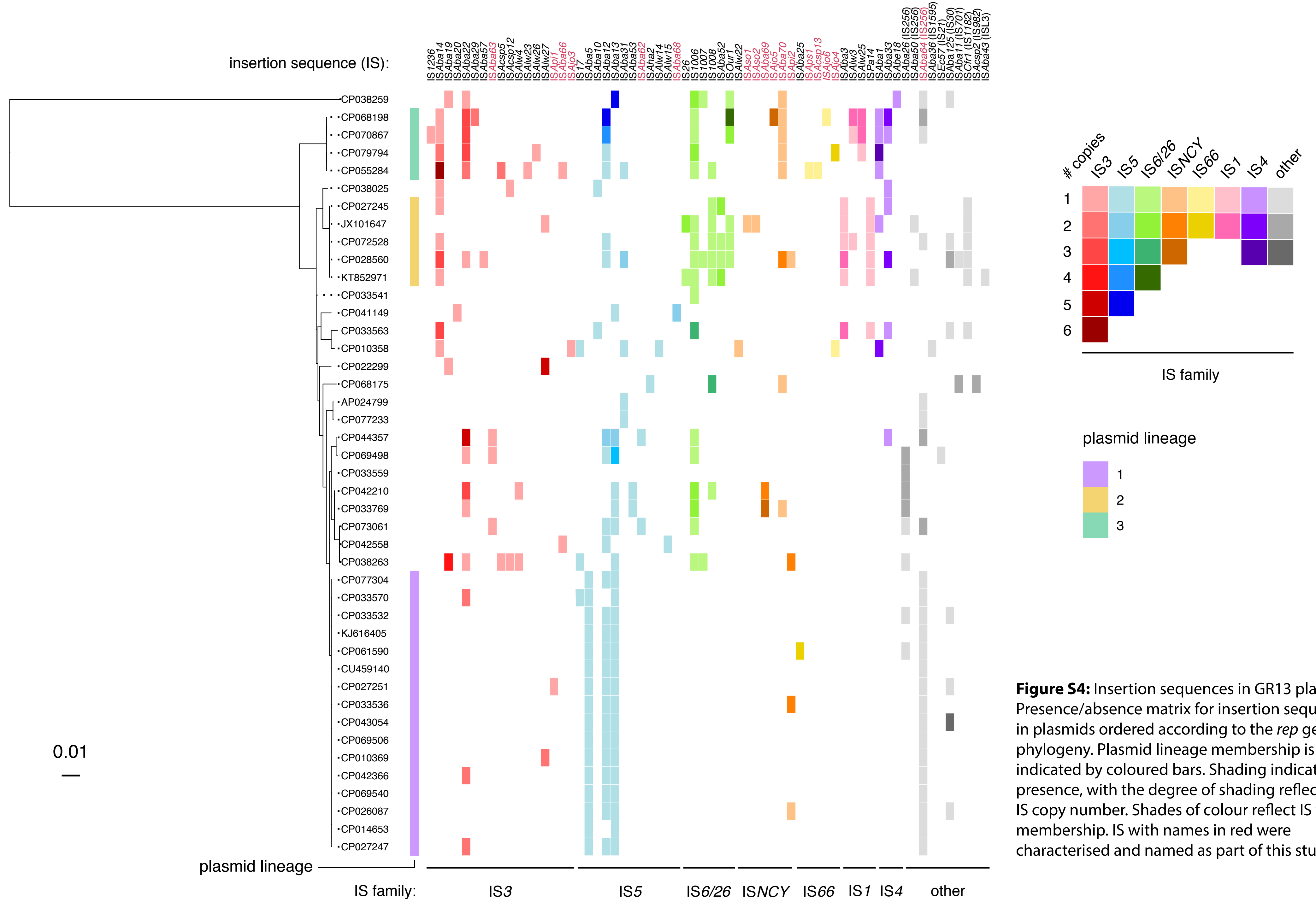

Figure S4: Insertion sequences in GR13 plasmids. Presence/absence matrix for insertion sequences in plasmids ordered according to the *rep* gene phylogeny. Plasmid lineage membership is indicated by coloured bars. Shading indicates IS presence, with the degree of shading reflective of IS copy number. Shades of colour reflect IS family membership. IS with names in red were characterised and named as part of this study.

Table S1: Primers used to detect DETAB-E227 plasmid replicons via PCR

Replicon type	Plasmid	Primer name	Primer sequence (5'-3')	Expected product size (bp)
GR24	pDETAB4	Hgz_103-F	TGGCAAGATTGAGGTGGTTC	327
		Hgz_103-R	AAGTTGGTCATATCCGTACTTTCG	
GR13	pDETAB5	GR13-F	TAGTAACCGTCTGATTAGAC	680
		GR13-R	GACCTTTCTTGATGGTATCG	

All primers were used in PCRs with annealing temperatures of 55°C.

Table S2: Novel IS found in the GR13 plasmid collection

IS	Closest match (family)	% ID	Plasmid
IS*Acsp13*	IS*Aba49* (IS*66*)	93.7	CP055284
IS*Aba62*	IS*Aba12* (IS*5*)	92.6	pDETAB13
IS*Aba63*	IS*Alw4* (IS*3*)	94.5	pDETAB13
IS*Aba64*	IS*Aba26* (IS*256*)	86.2	pDETAB13
IS*Aba66*	IS*Aba29* (IS*3*)	86.7	CP042558
IS*Aba67*	IS*Acra1* (IS*1595*)	88.0	CP042558
IS*Aba68*	IS*Aha1* (IS*5*)	92.3	CP041149
IS*Aba69*	IS*Alw22* (IS*NCY*)	91.0	CP033769
IS*Aba70*	IS*Ajo2* (IS*NCY*)	92.3	CP038259
IS*Ajo3*	IS*Aca1* (IS*3*)	90.8	CP010358
IS*Ajo4*	IS*Alw16* (IS*66*)	94.2	CP079794
IS*Ajo5*	IS*Aba32* (IS*NCY*)	92.8	CP068198
IS*Ajo6*	IS*Aba46* (IS*66*)	93.8	CP068198
IS*Api1*	IS*Acsp3* (IS*3*)	94.2	CP027251
IS*Api2*	IS*Ajo2* (IS*NCY*)	89.7	CP033536
IS*Aps1*	IS*Aba17* (IS*66*)	88.9	CP055284
IS*Aso2*	IS*Ajo2* (IS*NCY*)	93.5	JX101647
IS*Aso1*	IS*Alw22* (IS*NCY*)	89.3	JX101647

Table S3: Antibiotic minimum inhibitory concentrations

Isolates	Antibiotic[1] minimum inhibitory concentration (mg/L)[2]								
	IMP	MEM	CAZ	GEN	TOB	LEV	CIP	COL	TGC
DETAB-P39	0.125 (S)	0.5 (S)	4 (S)	0.5 (S)	1 (S)	0.06 (S)	0.125 (S)	1 (S)	0.25 (S)
DETAB-E227	256 (R)	128 (R)	>256 (R)	256 (R)	16 (R)	4 (I)	4 (R)	1 (S)	0.5 (S)
ATCC17978	0.5 (S)	0.25 (S)	4 (S)	0.5 (S)	0.5 (S)	0.125 (S)	0.125 (S)	0.5 (S)	0.5 (S)
ATCC17978 (pDETAB5)[3]	256 (R)	256 (R)	>256 (R)	>256 (R)	8 (I)	0.125 (S)	0.125 (S)	0.5 (S)	0.25 (S)

[1] IMP = imipenem, MEM = meropenem, CAZ = ceftazidime, GEN = gentamicin, TOB = tobramycin, LEV = levofloxacin, CIP = ciprofloxacin, COL = colistin, TGC = tigecycline

[2] minimum inhibitory concentrations classed as resistant (R), intermediate (I) or sensitive (S)

[3] transconjugant derived from mating DETAB-E227 with ATCC 17978

Table S4: Transfer frequency of pDETAB5 from DETAB-E227 to ATCC 17978

	Experiment			
	1	2	3	Mean
#Donors (D)	$1.90{\times}10^{9}$	$1.80{\times}10^{9}$	$4.2{\times}10^{9}$	
#Transconjugants (TC)	$1.75{\times}10^{3}$	$1.65{\times}10^{3}$	$1.05{\times}10^{3}$	
Conjugation frequency (TC/D)	$9.21{\times}10^{-7}$	$9.17{\times}10^{-7}$	$2.50{\times}10^{-7}$	$6.96{\times}10^{-7}$

Table S5: GR13 plasmids in GenBank (last search August 9, 2021)

Plasmid	Accession	Host	Country	Year	Source	Size	L	+GR	antibiotic resistance genes
p3ABAYE	CU459140	*A. baumannii*	France	2001	human clinical	94,413	1	-	-
pMS32-1	KJ616405	*A. pittii*	China; Taiwan	-	-	94,418	1	-	-
p6411-89.111kb	CP010369	*A. nosocomialis*	Colombia	2012	-	89,111	1	-	-
unnamed2	CP014653	*Acinetobacter* sp.	China; Panjin	2015	marine sediment	50,047	1	-	-
p2012N21-164-1	CP033536	*A. pittii*	China; Taiwan	2012	human	97,329	1	-	-
pC54_002	CP042366	*A. pittii*	Australia	2014	human clinical	76,008	1	-	-
p1_100020	CP027251	*A. pittii*	China; Chengdu	2015	human	77,340	1	-	-
p1_100004	CP027247	*A. pittii*	China; Chengdu	2015	human	66,765	1	-	-
pAP43-2	CP043054	*A. pittii*	China; Hangzhou	2018	human urine	92,276	1	-	-
unnamed3	CP069540	*A. pittii*	Germany	≤1994	human urine	94,387	1	-	-
unnamed2	CP069506	*A. pittii*	unknown	≤1994	human sputum	94,379	1	-	-
pAS61-1	CP061590	*A. seifertii*	China; Taiwan	2010	human blood	93,205	1	-	-
p2014N21-145-2	CP033570	*A. pittii*	China; Taiwan	2014	human	72,034	1	-	-
p2014S07-126-2	CP033532	*A. pittii*	China; Taiwan	2014	human	96,775	1	-	-
unnamed1	CP077304	*A. pittii*	Germany	≤1994	human ear discharge	94,386	1	-	-
p1_005069	CP026087	*A. pittii*	China; Chengdu	-	human	91,563	1	-	-
pDETAB5	CP072528	*A. baumannii*	China; Hangzhou	2019	intensive care unit environment	97,035	2	-	*bla*$_{\text{NDM-1}}$, *ble*$_{\text{MBL}}$, *bla*$_{\text{OXA-58}}$, *aacC2d*, *msr*(E)-*mph*(E), *sul2*
pM131-2	JX101647	*A. soli*	China; Taiwan	2010	human sputum	84,995	2	-	*bla*$_{\text{OXA-58}}$, *aphA1*, *aacC2d*, *sul2* (x2)
p255n_1	KT852971	*A. baumannii*	Vietnam	2005	human nasal	92,939	2	-	*bla*$_{\text{OXA-58}}$, *aacC2d*, *bla*$_{\text{VEB}}$, *arr-2*, *aadA1*, *aadB*, *cmlA6*, *msr*(E)-*mph*(E), *sul1*, *sul2*
pNDM1_010045	CP028560	*Acinetobacter* sp.	China; Chengdu	2015	sewage	190,170	2	34	*bla*$_{\text{NDM-1}}$, *ble*$_{\text{MBL}}$, *bla*$_{\text{OXA-58}}$ (x2), *aacC2d*, *floR*, *msr*(E)-*mph*(E), *sul2*, *merA-2*
pOXA58_005078	CP027245	*A. baumannii*	China; Chengdu	-	human	70,509	2	-	*bla*$_{\text{OXA-58}}$, *aacC2d*, *floR*, *msr*(E)-*mph*(E)
pBspH2	CP055284	*Acinetobacter* sp.	USA	1986	soil	161,809	3	-	-
unnamed3	CP068198	*A. johnsonii*	The Netherlands	2008	spacecraft-associated clean room	206,659	3	-	-
plas1	CP070867	*A. johnsonii*	China; Shanghai	2018	bigeye tuna	149,408	3	-	-
pAJ_082-3	CP079794	*A. johnsonii*	Pakistan	2016	intensive care unit sink	155,957	3	-	-
unnamed1	CP033769	*A. baumannii*	USA	2016	human sputum	97,783	-	-	-
pTS134338	CP042210	*A. baumannii*	India	2005	soil	134,338	-	-	-
pOCUAc17-1	AP024799	*A. pittii*	Japan*	≤2021	human blood*	69,156	-	-	-
unnamed1	CP077233	*A. pittii*	Germany	≤1990	human wound swab	69,574	-	-	-
pEC_gr13	CP038263	*A. baumannii*	Czech Republic	2018	frozen turkey liver	128,013	-	-	-
pE47_002	CP042558	*A. baumannii*	Australia	2013	hospital environment	59,744	-	-	-
pDETAB13	CP073061	*A. baumannii*	China; Hangzhou	2019	human rectal	91,083	-	-	-
pXBB1-8	CP010358	*A. johnsonii*	China; Chengdu*	-	sewage*	117,483	-	-	-
pCUVET596	CP041149	*A. baumannii*	Thailand	2017	dog urine	82,016	-	-	-
p2012C01-137-2	CP033559	*A. nosocomialis*	China; Taiwan	2012	human	72,978	-	-	-
unnamed1	CP068175	*A. ursingii*	The Netherlands	2003	spacecraft-associated clean room	120,510	-	-	-
pAR3	CP038025	*A. radioresistans*	Chile	2008	soil	80,103	-	-	-
p2014S06-099-1	CP033541	*A. pittii*	China; Taiwan	2014	human	125,715	-	-	-
pIC001A	CP022299	*A. johnsonii*	Japan	2003	Tokyo Bay water	94,476	-	26	-
pCAM180A	CP044357	*A. baumannii*	Cambodia	2016	human oral	92,034	-	-	-
p2010S01-197-2	CP033563	*A. nosocomialis*	China; Taiwan	2010	human	92,044	-	-	*bla*$_{\text{OXA-58}}$ (x2), *aacC2d*, *sul2*
unnamed2	CP069498	*A. pittii*	Germany	≤1994	human blood	128,321	-	24	-
pEH_Gr13	CP038259	*A. baumannii*	Czech Republic	2018	human tracheal	135,229	-	-	-

* = uncertain, information derived from submitter information and expected publication titles included in GenBank entries

L = lineage defined here, plasmids assigned to lineages 1, 2 or 3 are shaded pink, blue or orange, respectively

+GR = plasmids contain an additional replicon of GR type #

Table S6: Characteristics of *dif* modules examined in this study

module	size (bp)	putative function or content	reference plasmid
oxa58	2,257	carbapenem resistance	pDETAB5
sul2	5,051	sulphonamide resistance	pDETAB5
msrE-mphE	2,950	macrolide resistance	pDETAB5
aacC2d	9,468	aminoglycoside resistance	pDETAB5
dif-696a	696	toxin-antitoxin	pDETAB5
dif-696b	696	toxin-antitoxin	pDETAB13
dif-823	823	toxin-antitoxin (AbkAB)	pDETAB5
dif-701	701	toxin-antitoxin (HigAB)	pDETAB5
dif-1491	1,491	toxin-antitoxin + unknown ORFs	pDETAB13
dif-2111	2,111	alcohol tolerance	pDETAB13
dif-6874	6,874	alcohol tolerance	AP024799
dif-7136	7,136	alcohol tolerance	CU459140
dif-28327	28,327	copper resistance	CP068175
dif-RND	9,903	RND efflux	pDETAB5
dif-7932	7,932	metabolism; possibly aromatic compound degradation	CP022299
dif-1769	1,769	sulphate permease	CP022299
dif-6620	6,620	small plasmid + mobilisation determinant	CP022299
dif-801	801	VRR-NUC domain protein	CP022299
dif-614	614	unknown	pDETAB5
dif-1209	1,209	unknown	pDETAB5
dif-1612	1,612	unknown	pDETAB13
